# Transpancreatic precut sphincterotomy during ERCP: Learnability and outcomes across operator volumes and experience

**DOI:** 10.1055/a-2858-0520

**Published:** 2026-04-28

**Authors:** Wei-Chih Su, Chih-Hsiang Chen, Chia-Chi Wang, Tsung-Hsien Hsiao, Hung-Da Chen, Tzu-Hsiang Kung, Jiann-Hwa Chen

**Affiliations:** 1 Department of Gastroenterology 145204 Taipei Tzu Chi HospitalNew Taipei CityTaiwan; 2 School of Medicine 59216 Tzu Chi UniversityHualienTaiwan

**Keywords:** Pancreatobiliary (ERCP/PTCD), ERC topics, Quality and logistical aspects, Performance and complications, Training

## Abstract

**Background and study aims:**

Transpancreatic precut sphincterotomy (TPS) is an advanced option for difficult biliary cannulation during endoscopic retrograde cholangiopancreatography (ERCP) and is thought to be easier to learn than other techniques. We assessed TPS learnability across operators with differing volumes and experience levels.

**Patients and methods:**

TPS was introduced in April 2016. We retrospectively analyzed ERCPs performed thereafter (April 2016-March 2023) by three endoscopists: high-volume senior (~80/year), low-volume senior (~30/year), and low-volume newly independent (~30/year; since October 2020). Outcomes were successful common bild duct cannulation after TPS; cannulation/total ERCP times; and adverse events, including post-ERCP pancreatitis (PEP).

**Results:**

Among 539 ERCPs in patients with naïve papillae, TPS was used in 69 procedures (12.8%; 46, 16, and 7 for the high-volume senior, low-volume senior, and newly independent operators, respectively). Cannulation success after TPS was uniformly high (95.7%, 100%, and 100%, respectively). PEP occurred in 8.7%, 6.3%, and 28.6%, respectively, all mild. The newly independent operator had longer cannulation times (median [interquartile range] 35 [15] min vs. 22 [17] and 25 [12] min; *P* = 0.016) and total ERCP times (54 [42] vs. 42 [19] and 49 [32] min; *P* = 0.013). In multivariable analysis, older age was associated with lower PEP odds (odds ratio [OR] 0.936; *P* = 0.033), whereas end-stage renal disease (OR 47.433; *P* = 0.024) and procedures performed by the newly independent operator (OR 14.879; *P* = 0.028) were associated with higher odds.

**Conclusions:**

TPS is a readily adoptable advanced option for endoscopists proficient in basic ERCP skills. Newly independent operators can achieve comparable cannulation success but demonstrate early-proficiency patterns that may inform training, supervision, and procedure standardization.

## Introduction

Endoscopic retrograde cholangiopancreatography (ERCP) is a widely used procedure for diagnosis and treatment of biliary tract disorders, including stone extraction, stenting, and tissue sampling. Selective bile duct cannulation is fundamental to successful biliary intervention. However, the average primary cannulation rate with standard techniques is approximately 77.3% to 83.6% 1 . Cannulation difficulty is influenced by papillary morphology 2 and presence of a periampullary diverticulum 2 3 and surgically altered anatomy 4 . Operator experience and practice volume also affect outcomes 5 ; frequency of difficult cannulation is correlated with years of endoscopist experience and center volume 2 , with lower volumes (< 200 ERCPs per year) being associated with higher complication rates 6 . More cannulation attempts and longer cannulation time are positively correlated with post-ERCP pancreatitis (PEP) 7 . The European Society of Gastrointestinal Endoscopy (ESGE) defines difficult cannulation as a cannulation time exceeding 5 minutes 8 .

When standard cannulation fails, needle-knife precut sphincterotomy is the most commonly recommended rescue option. Although it can improve access, the higher rate of success with this procedure may be accompanied by more adverse events (AEs), with PEP reported in up to 14% of patients 9 . Moreover, its learning curve 10 and dependence on operator and institutional volume 11 further limit its widespread adoption. In lower-volume settings, especially for novice ERCP endoscopists, a more accessible and safer approach is desirable.

Transpancreatic precut sphincterotomy (TPS) resembles standard wire-guided biliary sphincterotomy but is performed over a guidewire placed in the pancreatic duct. By incising the septum between the pancreatic and bile ducts, the biliary orifice can be exposed along the left cutting edge, facilitating selective cannulation. Compared with other advanced techniques, TPS is often considered technically less complex and easier to learn 12 . However, although TPS has been reported to improve cannulation outcomes in lower-volume settings 13 , learning characteristics of TPS remain insufficiently studied.

Our center performs approximately 200 therapeutic ERCPs annually. TPS was introduced in April 2016 and subsequently became our primary strategy for managing difficult cannulations. A newly trained endoscopist joined the staff in October 2020 after completing fellowship training at another ERCP center. This study aimed to evaluate learnability of TPS from the time of its introduction by comparing outcomes for difficult bile duct cannulation in patients with a naïve papilla across three operators with differing levels of experience and case volumes: a high-volume senior endoscopist, a low-volume senior endoscopist, and a newly independent, low-volume endoscopist. The primary outcome was successful common bile duct cannulation after TPS; secondary outcomes included cannulation time, total ERCP time, and AEs (particularly PEP).

## Patients and methods

### Study design and data collection

TPS was introduced at our hospital in April 2016. We retrospectively identified therapeutic ERCP procedures in patients with a naïve papilla in which TPS was attempted from April 2016 through March 2023. Three attending endoscopists were included. Endoscopist 1 (a high-volume senior operator) performed approximately 80 ERCP procedures per year and had 21 years of ERCP experience at the start of data collection as of April 2016. Endoscopist 2 (a low-volume senior operator) performed approximately 20 to 30 ERCP procedures per year and had 12 years of ERCP experience as of April 2016. Endoscopist 3 (a low-volume, newly independent operator) performed approximately 20 to 30 ERCP procedures per year and had begun independently performing ERCP in October 2020. Accordingly, ERCP data were collected from April 2016 for Endoscopists 1 and 2 and from October 2020 for Endoscopist 3. We extracted patient age, sex, clinical information, ERCP findings, and procedure outcomes. ERCP indications were categorized as biliary stones, distal common bile duct obstruction, proximal bile duct obstruction, and other. The ampulla of Vater was classified according to Haraldsson’s system as Type 1 (regular), Type 2 (small; diameter not exceeding 3 mm), Type 3 (enlarged or protruding), or Type 4 (creased or ridged) 14 . Periampullary diverticula were classified according to the method of Shi et al. 15 . Papilla and diverticulum classifications were determined retrospectively from stored duodenoscopic images. This study was approved by the Institutional Review Board (14-IRB145) of Taipei Tzu Chi Hospital, Buddhist Tzu Chi Medical Foundation.

### ERCP procedure

**Fig. 1:**
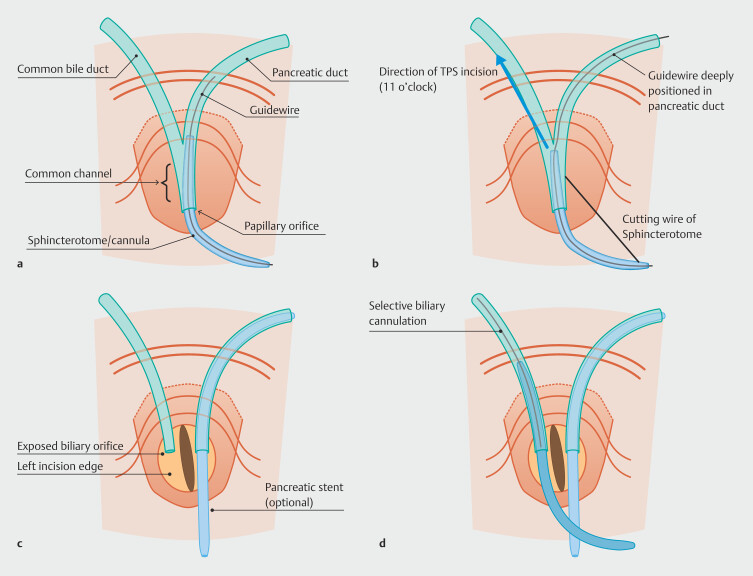
Procedure sequence of wire-guided transpancreatic sphincterotomy (TPS). **a** Unintended pancreatic duct cannulation with guidewire placement during difficult biliary cannulation. **b** Advancement of the guidewire deeply into the pancreatic duct followed by positioning of a standard sphincterotome over the pancreatic guidewire. Transpancreatic sphincterotomy is performed in the 11 o’clock direction along the pancreaticobiliary septum. **c** Post-incision configuration of the papilla demonstrating exposure of the incision edges and visualization of the exposed biliary orifice along the left aspect of the incision. Placement of a pancreatic stent may be performed at this step at operator discretion. **d** Selective biliary cannulation through the exposed biliary orifice into the common bile duct.

All ERCPs were performed under monitored anesthesia care. The initial approach was standard guidewire-assisted biliary cannulation. When selective common bile duct cannulation was not achieved within 5 minutes, the cannulation strategy was reassessed. TPS was preferentially performed in cases of unintended pancreatic duct cannulation when deep guidewire access to the pancreatic duct was achievable. Needle-knife precut was considered when pancreatic duct access could not be obtained. The decision to proceed with TPS and timing of its initiation were determined at endoscopist discretion. TPS was performed as illustrated in Fig. 1 . A guidewire was advanced deeply into the pancreatic duct and a standard sphincterotome was positioned over the pancreatic guidewire. Wire-guided transpancreatic sphincterotomy was then performed in the 11 o’clock direction along the pancreaticobiliary septum. After the incision was completed, a pancreatic stent (4–5F, 5 cm) was generally placed; however, in selected cases, stent placement was performed prior to incision at endoscopist discretion. Biliary cannulation was subsequently attempted along the left margin of the incision. Pharmacological prophylaxis against PEP consisted of preprocedure hydration with 1000 mL lactated Ringer’s solution infused over 8 hours, initiated 15 minutes before ERCP, plus rectal nonsteroidal anti-inflammatory drugs (NSAIDs) (100 mg diclofenac); this protocol was first implemented in November 2018.

### Definitions and data measurement

Bile duct cannulation time, total ERCP time, and post-ERCP AEs (immediate bleeding requiring endoscopic therapy, delayed bleeding, PEP, cholangitis, and perforation) were recorded. During ERCP, endoscopic images were routinely captured on arrival to the second portion of the duodenum with an en face view of the papilla and at time of successful deep cannulation; fluoroscopic images were obtained at time of deep ductal guidewire insertion and at the end of the examination to assess for perforations. Cannulation time and total ERCP time were determined by reviewing timestamps on endoscopic and cholangiographic images. Cannulation time was measured from first visualization of the papilla to successful cannulation of the common bile duct and total ERCP time was measured from the first visualization of the papilla to the end of the examination. Difficult cannulation was defined as cannulation time exceeding 5 minutes. PEP was defined as clinical pancreatitis diagnosed by typical upper abdominal pain plus a lipase level at least three times the upper limit of normal more than 24 hours after the procedure, requiring a prolonged hospital stay with severity graded according to the Cotton criteria 16 . Cholangitis was defined as fever >38 °C with a biliary source and without acute cholecystitis. Delayed bleeding was defined as clinical gastrointestinal bleeding (melena, hematemesis, or a decrease in hemoglobin concentration ≥2 g/dL) or need for transfusion after the procedure. ERCP-related perforations were limited to sphincterotomy-related perforations, identified by extraluminal air on radiological imaging. Two experienced endoscopists (W-C Su and T-H Hsiao) independently classified papilla and diverticulum types and measured all time intervals; discrepancies were resolved by consensus with a third endoscopist (J-H Chen).

### Statistical analysis

Categorical variables were summarized as counts and percentages. Continuous variables were summarized as mean ± standard deviation when approximately normally distributed or as median and interquartile range (IQR) otherwise. Categorical variables were compared using the chi-square test or Fisher’s exact test, as appropriate. For continuous variables, Student’s *t* test or one-way ANOVA was used when distributional and variance assumptions were reasonably met (Welch’s correction was applied when variances were unequal); otherwise, the Mann–Whitney U test (two groups) or the Kruskal–Wallis test (≥3 groups) was used. Factors associated with PEP were examined using logistic regression analysis; for events with low counts, Firth’s penalized logistic regression was applied. Models also were adjusted for case-level prophylaxis use (rectal NSAIDs and lactated Ringer’s hydration) in light of the protocol introduced in November 2018. Statistical analyses were performed using the Statistical Package for the Social Sciences (SPSS) version 27.0 for Windows (SPSS Inc., Chicago, Illinois, United States. All *P* values were two-tailed; *P* <0.05 was considered to indicate statistical significance.

## Results

During the study period, the three endoscopists performed 539 therapeutic ERCPs in patients with a naïve papilla. As summarized in Table 1 , 267 patients (49.5%) were male and mean age was 68.4 ± 16.0 years. Biliary stones were the most common indication for ERCP (83.1%), followed by distal (10.0%) and proximal (6.7%) biliary obstruction; other indications (2.0%) included postoperative bile leakage in seven patients, hepatocellular carcinoma-related hemobilia in two patients, and suspected sphincter of Oddi dysfunction and bile duct obstruction due to chronic pancreatitis in one patient each. On macroscopic inspection, the papilla was most often Haraldsson type 1 (36.9%), followed by types 3 (27.3%), 2 (15.4%), and 4 (10.4%). Periampullary diverticula were present in approximately 40% of patients, most commonly type IIb (16.3%), type III (10.9%), type IIa (7.4%), and type I diverticula (1.5%). Baseline characteristics were similar across endoscopists, except for prevalence of end-stage renal disease (ESRD) ( *P* = 0.037). Among all ERCP procedures, 300 of 539 (55.7%) had a cannulation time longer than 5 minutes. Proportions of difficult cannulation were 56.0%, 48.3%, and 73.2% for Endoscopists 1, 2, and 3, respectively ( *P* = 0.006). Approximately one-quarter of the procedures required an advanced cannulation technique; among them, TPS was the most frequently used (12.8%), followed by pancreatic stent-assisted cannulation (6.9%), needle-knife precut (4.6%), and the double-guidewire technique (0.7%). The proportion of cases in which TPS was used did not differ markedly among the endoscopists (13.7%, 10.9%, and 12.5%, respectively).

**Table:** **Table 1** Baseline characteristics of ERCP procedures by endoscopist in patients with naïve papillae.

	All (n = 539)	Endoscopist 1 (n = 336)	Endoscopist 2 (n = 147)	Endoscopist 3 (n = 56)	*P* value
**Male**	267 (49.5%)	168 (50.0%)	71 (48.3%)	28 (50.0%)	0.940 ^§^
Age ^*^	68.4 (16.0)	67.8 (15.8)	70.2 (16.6)	66.5 (15.6)	0.208 ^¶^
**Indication**					0.276 ^§^
Biliary stone	438 (83.1%)	269 (80.1%)	120 (81.6%)	49 (87.5%)	
Distal obstruction	54 (10.0%)	40 (11.9%)	11 (7.5%)	3 (5.4%)	
Proximal obstruction	36 (6.7%)	20 (6.0%)	14 (9.5%)	2 (3.6%)	
Other	11 (2.0%)	7 (2.1%)	2 (1.4%)	2 (3.6%)	
**History of biliary pancreatitis**	67 (12.4%)	40 (11.9%)	20 (13.6%)	7 (12.5%)	0.873 ^§^
**ESRD**	23 (4.3%)	12 (3.6%)	11 (7.5%)	0 (0%)	0.037 ^§^
** Macroscopic appearance of the ampulla of Vater ^†^**	0.089 ^§^				
Type 1	199 (36.9%)	128 (38.1%)	47 (32.0%)	24 (42.9%)	
Type 2	83 (15.4%)	48 (14.3%)	39 (19.7%)	6 (10.7%)	
Type 3	147 (27.3%)	93 (27.7%)	45 (30.6%)	9 (16.1%)	
Type 4	110 (20.4%)	67 (19.9%)	26 (17.7%)	17 (30.4%)	
** Periampullary diverticulum ^‡^**	0.495 ^§^				
No diverticulum	344 (63.8%)	217 (64.6%)	93 (63.3%)	34 (60.7%)	
Type I	8 (1.5%)	6 (1.8%)	2 (1.4%)	0 (0%)	
Type IIa	40 (7.4%)	19 (5.7%)	17 (11.6%)	4 (7.1%)	
Type IIb	88 (16.3%)	55 (16.4%)	22 (15.0%)	11 (19.6%)	
Type III	59 (10.9%)	39 (11.6%)	13 (8.8%)	7 (12.5%)	
Difficult cannulation	300 (55.7%)	188 (56.0%)	71 (48.3%)	41 (73.2%)	0.006 ^§^
**Cannulation method**		0.322 ^§^			
Standard	404 (75.0%)	243 (72.3%)	121 (82.3%)	40 (71.4%)	
TPS	69 (12.8%)	46 (13.7%)	16 (10.9%)	7 (12.5%)	
Double guidewire	4 (0.7%)	4 (1.2%)	0 (0%)	0 (0%)	
Needle-knife precut	25 (4.6%)	18 (5.4%)	3 (2.0%)	4 (7.1%)	
Pancreatic stent-assisted	37 (6.9%)	25 (7.4%)	7 (4.8%)	5 (8.9%)	
Data are presented as the mean (standard deviation) or number (%), unless otherwise specified. ERCP, endoscopic retrograde cholangiopancreatography; ESRD, end-stage renal disease; TPS, transpancreatic precut sphincterotomy. *Mean (standard deviation). ^†^ Type 1: regular papilla; Type 2: small papilla, often flat, with a diameter ≤ 3 mm; Type 3: protruding or pendulous papilla; Type 4: creased or ridged papilla. ^‡^ Papilla located completely inside the diverticulum (type I), papilla located in the inner (type IIa) and outer (type IIb) margins of the diverticulum, and papilla located outside the diverticulum (type III). ^§^ Derived from chi-square test. ^¶^ Derived from one-way ANOVA.					

**Fig. 2:**
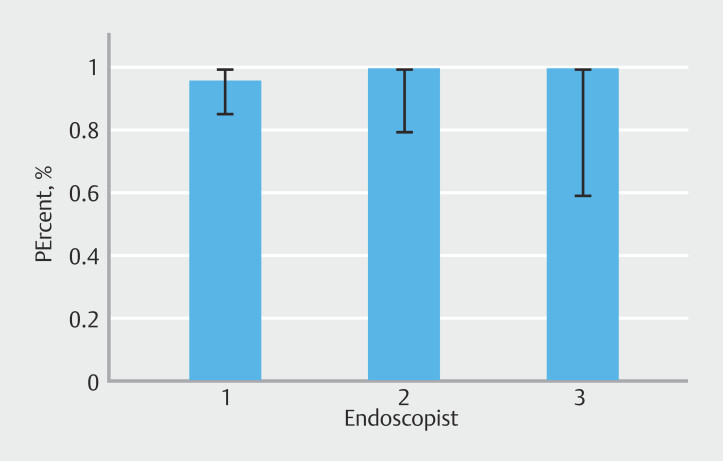
Successful common bile duct cannulation following transpancreatic sphincterotomy by an endoscopist. Points represent operator-specific proportions with exact binomial 95% confidence intervals (Clopper–Pearson). The numbers of TPS procedures were 46, 16, and 7 for Endoscopists 1, 2, and 3, respectively. For 100% proportions, confidence intervals extend only downward.

**Fig. 3:**
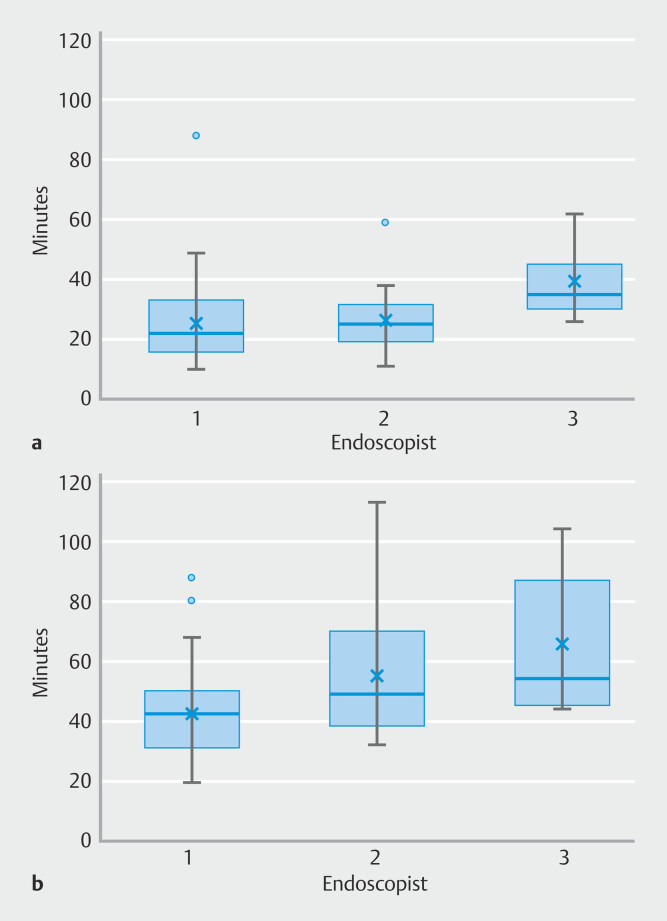
Cannulation and total ERCP times by endoscopist. **a** Cannulation time. **b** Total ERCP time. Box-and-whisker plots (median, IQR, whiskers 1.5×IQR) with overlaid points; minutes on the y-axis. Kruskal–Wallis across endoscopists: **a** *P* = 0.016 (A) and **b** *P* = 0.013.

As shown in Table 2 , among the 69 TPS procedures, Endoscopist 1 performed 46, Endoscopist 2 performed 16, and Endoscopist 3 performed 7. Patient and procedure characteristics, including age, sex, indication, history of biliary pancreatitis, ESRD, macroscopic appearance of the ampulla of Vater, or presence of a periampullary diverticulum, did not differ across endoscopists. Pharmacologic prophylaxis for PEP (hydration with lactated Ringer’s solution and rectal NSAIDs) was first implemented in November 2018; consequently, this measure was more frequently used in procedures performed by Endoscopist 3. The rate of successful common bile duct cannulation after TPS was high overall (Endoscopist 1, 95.7%; Endoscopist 2, 100%; Endoscopist 3, 100%; *P* = 0.598; Fig. 2 ). Endoscopists 2 and 3 achieved 100% cannulation (n = 16 and n = 7, respectively); the exact 95% lower bounds were 0.79 and 0.59. Rates of PEP were 8.7%, 6.3%, and 28.6% for Endoscopists 1, 2, and 3, respectively; however, the differences were not statistically significant according to a chi-square test ( *P* = 0.225). Adjusted associations for PEP are reported in Table 3 . Cannulation time and total ERCP time had right-skewed distributions (Shapiro–Wilk *P* < 0.001; skewness/standard error [SE] > 2) and are reported as the median (IQR). Median cannulation time was 22 (17), 25 (12), and 35 (15) minutes for Endoscopists 1, 2, and 3, respectively (Kruskal–Wallis *P* = 0.016). Median total ERCP time was 42 (19), 49 (32), and 54 (42) minutes, respectively (Kruskal–Wallis *P* = 0.013) ( Fig. 3 ). All PEP events were mild and no significant differences were observed in other post-ERCP complications.

**Table:** **Table 2** Clinical characteristics and outcomes of TPS procedures by endoscopist.

	All TPS (n = 69)	Endoscopist 1 (n = 46)	Endoscopist 2 (n = 16)	Endoscopist 3 (n = 7)	*P* value
**Male**	35 (50.7%)	26 (56.5%)	7 (43.8%)	2 (28.6%)	0.316 ^**^
Age ^*^	68.1 (16.4)	65.1 (17.4)	72.9 (11.6)	76.1 (15.2)	0.099 ^††^
**Indication**					0.385 ^**^
Biliary stone	50 (72.5%)	34 (73.9%)	10 (62.5%)	6 (85.7%)	
Distal obstruction	10 (14.5%)	8 (17.4%)	2 (12.5%)	0 (0%)	
Proximal obstruction	9 (13.0%)	4 (8.7%)	4 (25.0%)	1 (14.3%)	
History of biliary pancreatitis	10 (14.5%)	8 (17.4%)	0 (0%)	2 (28.6%)	0.126 ^**^
ESRD	2 (2.9%)	1 (2.2%)	1 (6.3%)	0 (0%)	0.627 ^**^
Macroscopic appearance of the ampulla of Vater ^†^	0.178 ^**^				
Type 1	22 (31.9%)	10 (21.7%)	8 (50.0%)	4 (57.1%)	
Type 2	6 (8.7%)	6 (13.0%)	0 (0%)	0 (0%)	
Type 3	22 (31.9%)	16 (34.8%)	5 (31.3%)	1 (14.3%)	
Type 4	19 (27.5%)	14 (30.4%)	3 (18.8%)	2 (28.6%)	
** Periampullary diverticulum ^‡^**	0.805 ^**^				
No diverticulum	46 (66.7%)	32 (69.6%)	10 (62.5%)	4 (57.1%)	
Type I	1 (1.4%)	1 (2.2%)	0 (0%)	0 (0%)	
Type IIa	4 (5.8%)	2 (4.3%)	1 (6.3%)	1 (14.3%)	
Type IIb	11 (15.9%)	7 (15.2%)	2 (12.5%)	2 (28.6%)	
Type III	7 (10.1%)	4 (8.7%)	3 (18.8%)	0 (0%)	
Post-ERCP pancreatitis prevention					
Pancreatic stenting	66 (95.7%)	43 (93.5%)	16 (100%)	7 (100%)	0.457 ^**^
Lactated Ringer’s solution hydration ^¶^	39 (56.5%)	25 (54.3%)	8 (50.0%)	6 (85.7%)	0.247 ^**^
Rectal NSAIDs ^¶^	26 (37.7%)	16 (34.8%)	4 (25.0%)	6 (85.7%)	0.017 ^**^
**ERCP outcomes**					
Successful biliary cannulation	67 (97.1%)	44 (95.7%)	16 (100%)	7 (100%)	0.598 ^**^
Cannulation time ^‡‡^	26.0 (17)	22.0 (17)	25.0 (12)	35.0 (15)	0.016 ^§§^
ERCP time ^‡‡^	46.0 (21)	42.0 (19)	49.0 (32)	54.0 (42)	0.013 ^§§^
Post-ERCP pancreatitis	7 (10.1%)	4 (8.7%)	1 (6.3%)	2 (28.6%)	0.225 ^**^
Bleeding requiring hemostasis	8 (11.6%)	6 (13.0%)	2 (12.5%)	0 (0%)	0.599 ^**^
Delayed bleeding	3 (4.3%)	3 (6.5%)	0 (0%)	0 (0%)	0.457 ^**^
Cholangitis	4 (5.8%)	2 (4.3%)	1 (6.3%)	1 (14.3%)	0.575 ^**^
Perforation	1 (1.4%)	1 (2.2%)	0 (0%)	0 (0%)	0.776 ^**^
Data are presented as the mean (standard deviation), median (interquartile range), or number (%), as appropriate. Cannulation and ERCP times are expressed as the median (interquartile range). ANOVA, analysis of variance; ERCP, endoscopic retrograde cholangiopancreatography; ESRD, end-stage renal disease; IQR, interquartile range; NSAID, nonsteroidal anti-inflammatory drug; TPS, transpancreatic precut sphincterotomy. ^*^ Mean (standard deviation). ^†^ Type 1: regular papilla; type 2: small papilla, often flat, with a diameter ≤ 3 mm; type 3: protruding or pendulous papilla; type 4: creased or ridged papilla. ^‡^ Papilla located completely inside the diverticulum (type I), papilla located in the inner (type IIa) or outer (type IIb) margin of the diverticulum, and papilla located outside the diverticulum (type III) ^§^ Lactated Ringer’s solution hydration and NSAID administration were implemented in November 2018. ^**^ Derived from chi-square test ^††^ Derived from ANOVA test. ^‡‡^ Median (IQR). ^§§^ Derived from Kruskal–Wallis test.					

Univariable and multivariable logistic regression analyses for PEP among TPS patients are summarized in Table 3 . In univariable analyses, none of the associations reached statistical significance. In the multivariable model, older age was associated with lower odds of PEP (per 1-year increase: odds ratio [OR] 0.936, 95% confidence interval [CI] 0.881–0.995; *P* = 0.033), ESRD was associated with higher odds of PEP (OR 47.433, 95% CI 1.660–1355.114; *P* = 0.024), and procedures performed by Endoscopist 3 (relative to those performed by Endoscopist 1) were associated with higher odds of PEP (OR 14.879, 95% CI 1.331–166.338; *P* = 0.028). Sex, history of biliary pancreatitis, indication, cannulation time, pancreatic stenting, hydration, and rectal NSAIDs were not significantly associated with the development of PEP. In models including rectal NSAIDs and hydration, neither measure was independently associated with PEP, and associations with age, ESRD, and operator were materially unchanged.

**Table:** **Table 3** Univariable and multivariable logistic regression analyses for post-ERCP pancreatitis among TPS cases.

Variable		Univariate analysis		Multivariate analysis	
		OR (95% CI)	*P* value	OR (95% CI)	*P* value
Age		0.965 (0.920–1.011)	0.131	0.936 (0.881–0.995)	0.033
Sex	Female	Reference			
	Male	0.750 (0.155–3.632)	0.721		
History of biliary pancreatitis	No	Reference			
	Yes	0.981 (0.105–9.144)	0.987		
ESRD	No	Reference		Reference	
	Yes	10.167 (0.652–184.011)	0.117	47.433 (1.660–1355.114)	0.024
Endoscopist	Endoscopist 1	Reference		Reference	
	Endoscopist 2	0.700 (0.072–6.770)	0.758		
	Endoscopist 3	4.200 (0.607–29.056)	0.146	14.879 (1.331–166.338)	0.028
Indication ^*^	Other indications	Reference			
	Biliary stone	6.72 (0.371–121.769)	0.197		
Cannulation time		0.988 (0.928–1.052)	0.707		
Pancreatic stenting ^*^	No	Reference			
	Yes	0.882 (0.027–29.400)	0.944		
Hydration	No	Reference			
	Yes	0.542 (0.112–2.629)	0.447		
NSAIDs	No	Reference			
	Yes	0.633 (0.114–3.528)	0.602		
Odds ratios are presented with 95% confidence intervals. Multivariable models were additionally adjusted for rectal NSAIDs and lactated Ringer’s hydration. Firth’s penalized logistic regression was applied for variables with sparse events, as indicated in the table. CI, confidence interval; ERCP, endoscopic retrograde cholangiopancreatography; ESRD, end-stage renal disease; NSAID, nonsteroidal anti-inflammatory drug; OR, odds ratio; TPS, ^*^ Derived from Firth’s penalized logistic regression.					

## Discussion

TPS relies on the same equipment and core skills as standard wire-guided biliary sphincterotomy. In our cohort, the two senior endoscopists achieved comparable TPS performance despite the difference in practice volumes. The newly independent endoscopist achieved a similar cannulation success rate but required longer cannulation and total procedure times and had a higher rate of PEP. To our knowledge, this is the first study to evaluate the learnability of TPS at the operator level from the time of its introduction while directly comparing outcomes across endoscopists with distinct procedure volumes and experience levels. These data align with the working premise that TPS is readily adoptable once core ERCP skills are established.

Among the three ESGE-recommended advanced cannulation strategies 7 , TPS is often considered less technically complex and more readily adopted. In contrast, freehand needle-knife precut has a steeper learning curve, with success and safety closely linked to operator and center procedure volume 10 17 18 , and the double-guidewire technique tends to yield lower rates of cannulation success 19 while adding procedural complexity, potentially reducing stone clearance rates 20 . In our series, when TPS was first implemented by the two senior endoscopists with different annual ERCP volumes, their cannulation success rates, cannulation times, total ERCP times, and ERCP-related complication rates were similar, suggesting that TPS performance among experienced endoscopists is not materially influenced by their practice volumes. Our findings support the use of TPS as an advanced cannulation technique that can be readily deployed, as needed, by endoscopists who are already proficient in basic ERCP skills.

Compared with routine endoscopy, learning ERCP cannulation requires the simultaneous control of a side-viewing duodenoscope, an elevator, a cannula, and a guidewire and therefore typically demands a higher practice volume 21 ; however, newly independent endoscopists, by definition, lack sufficient practice volume. In our series, the newly independent endoscopist achieved a cannulation success rate comparable to that of the senior operators but with longer cannulation and total ERCP times and a higher rate of PEP. These operator-level differences persisted after adjustment for rectal NSAIDs and hydration, suggesting that prophylaxis use does not explain the observed association. These differences are consistent with early-proficiency effects and may improve as experience accrues and standardized prophylaxis is consistently applied. These findings support the selective use of TPS as a rescue technique in independent practice, alongside standardized PEP prophylaxis and timely involvement of a senior endoscopist if cannulation becomes prolonged. The findings also suggest that TPS could be incorporated into structured ERCP training curricula, with appropriate supervision during early independent practice.

Despite its practical advantages, TPS has two relevant limitations. First, TPS presupposes guidewire access to the pancreatic duct; when neither duct is accessible, freehand needle-knife precut remains necessary as part of comprehensive ERCP practice. However, when TPS is available, the cannulation strategy can be more permissive toward pancreatic wire placement because inadvertent pancreatic cannulation can be deliberately converted into transpancreatic septotomy, thereby reframing a traditionally avoided event as a controlled method for achieving selective biliary access with a high likelihood of success. Second, concerns persist regarding potential long-term pancreatic duct injury after septotomy. A single-center series of 369 TPS procedures (2005–2016) reported no pancreatic duct strictures over 11 years of experience 12 , and a long-term follow-up of 143 patients (median 6 years; range 4–10 years) revealed no cases of chronic pancreatitis after TPS 22 . In our center, since the adoption of TPS in 2016, we have not observed long-term complications related to TPS; nevertheless, larger studies with systematic follow-up are warranted to confirm its long-term safety.

Our study has the following strengths. First, this study is among the first to evaluate the learnability of TPS from the time of its introduction and to compare the performance of operators with distinct levels of experience and case volumes (e.g., a high-volume senior, a low-volume senior, and a low-volume, newly independent operator), thereby providing practice-relevant evidence. Second, consecutive case capture within a single program and a shared training/procedural workflow minimized interinstitutional variability and improved internal consistency. However, several limitations merit consideration. First, the retrospective, single-center design introduces potential selection bias in deciding when to proceed to TPS; although the TPS utilization rates were similar across endoscopists, residual selection bias cannot be excluded. Second, the analytic sample was limited (69 TPS sessions with 7 PEP events), yielding wide CIs and imprecise estimates. Although newly independent operators rarely accrue high case volumes, these numbers reflect early independent practice from the real world. In addition, we did not formally define a numeric case threshold required to achieve TPS proficiency, nor did we perform a dedicated learning curve analysis. The consistently high cannulation success observed from early TPS cases limited our ability to evaluate temporal performance changes. Importantly, all the participating endoscopists were attending physicians with established ERCP experience at time of TPS adoption; therefore, our findings should not be extrapolated to trainees or endoscopists without prior ERCP competence. Nevertheless, the observed early stability in terms of TPS outcomes suggests that adoption of TPS may be feasible among endoscopists who are already proficient in core ERCP techniques, although these observations should be interpreted cautiously in the absence of formal learning curve analysis. Future prospective studies with larger case numbers and formal learning curve analyses are warranted to better define TPS proficiency thresholds and to assess generalizability across different training levels and practice settings. Third, period effects may have confounded operator comparisons, as prophylactic hydration and rectal NSAIDs were implemented institutionally in November 2018, and the newly independent endoscopist began independent practice in October 2020. In multivariable models, neither hydration nor rectal NSAIDs were independently associated with PEP, but residual confounding effects cannot be excluded.

## Conclusions

TPS provides high-quality access in cases of difficult cannulation and is readily adoptable across operators; beginners and low-volume endoscopists can achieve comparable success rates to those of senior and high-volume endoscopists, albeit with longer procedure times and higher PEP rates.
